# Lycophyte plastid genomics: extreme variation in GC, gene and intron content and multiple inversions between a direct and inverted orientation of the rRNA repeat

**DOI:** 10.1111/nph.15650

**Published:** 2019-01-24

**Authors:** Jeffrey P. Mower, Peng‐Fei Ma, Felix Grewe, Alex Taylor, Todd P. Michael, Robert VanBuren, Yin‐Long Qiu

**Affiliations:** ^1^ Center for Plant Science Innovation University of Nebraska Lincoln NE 68588 USA; ^2^ Department of Agronomy and Horticulture University of Nebraska Lincoln NE 68583 USA; ^3^ Germplasm Bank of Wild Species Kunming Institute of Botany Chinese Academy of Sciences Kunming Yunnan 650201 China; ^4^ Grainger Bioinformatics Center, Science and Education Field Museum of Natural History Chicago IL 60605 USA; ^5^ Department of Ecology and Evolutionary Biology University of Michigan Ann Arbor MI 48109 USA; ^6^ J. Craig Venter Institute La Jolla CA 92037 USA; ^7^ Department of Horticulture Michigan State University East Lansing MI 48824 USA

**Keywords:** evolutionary stasis, gene loss, inversion, *Isoetes* (quillworts), Lycopodiaceae (clubmosses), Lycopodiophyta (lycophytes), plastid genome (plastome), *Selaginella* (spikemosses)

## Abstract

- Lycophytes are a key group for understanding vascular plant evolution. Lycophyte plastomes are highly distinct, indicating a dynamic evolutionary history, but detailed evaluation is hindered by the limited availability of sequences.
- Eight diverse plastomes were sequenced to assess variation in structure and functional content across lycophytes.
- Lycopodiaceae plastomes have remained largely unchanged compared with the common ancestor of land plants, whereas plastome evolution in *Isoetes* and especially *Selaginella* is highly dynamic. *Selaginella* plastomes have the highest GC content and fewest genes and introns of any photosynthetic land plant. Uniquely, the canonical inverted repeat was converted into a direct repeat (DR) via large‐scale inversion in some *Selaginella* species. Ancestral reconstruction identified additional putative transitions between an inverted and DR orientation in *Selaginella* and *Isoetes* plastomes. A DR orientation does not disrupt the activity of copy‐dependent repair to suppress substitution rates within repeats.
- Lycophyte plastomes include the most archaic examples among vascular plants and the most reconfigured among land plants. These evolutionary trends correlate with the mitochondrial genome, suggesting shared underlying mechanisms. Copy‐dependent repair for DR‐localized genes indicates that recombination and gene conversion are not inhibited by the DR orientation. Gene relocation in lycophyte plastomes occurs via overlapping inversions rather than transposase/recombinase‐mediated processes.

## Introduction

Across the diversity of land plants, the plastid genome (plastome) is distinguished by its overall conservation in size, structure and content. Plastomes of photoautotrophic land plants typically range from 120 to 160 kb in length, with *c*. 80 protein‐coding genes, four rRNAs, and *c*. 30 tRNAs arranged into a structure that usually includes large and small single‐copy (SC) regions (termed LSC and SSC) separated by a large inverted repeat (IR) in two copies (Wicke *et al*., 2011; Jansen & Ruhlman, 2012; Mower & Vickrey, 2018). One of the most notable effects of this genomic structure is that substitution rates are several times lower in the IR relative to SC regions (Wolfe *et al*., 1987; Perry & Wolfe, 2002; Li *et al*., 2016; Zhu *et al*., 2016). This reduction in IR substitution rates has been attributed to a copy‐dependent repair mechanism such as biased gene conversion (Birky & Walsh, 1992), which may be facilitated by frequent intramolecular recombination between IR copies that produces two isomeric forms of the plastome (Bohnert & Loffelhardt, 1982; Palmer, 1983).

Comparative analysis of plastome structures from diverse land plant representatives demonstrated that inversions and IR boundary shifts (via expansion or contraction) are the primary processes of structural rearrangements, with varying frequency among lineages (reviewed in Mower & Vickrey, 2018). More rarely, a third type of rearrangement (variously termed transposition or translocation) has been invoked to explain the intramolecular relocation of one or more genes within the plastome (e.g., Milligan *et al*., 1989; Cosner *et al*., 1997, 2004; Chumley *et al*., 2006; Tsuji *et al*., 2007; Karol *et al*., 2010; Knox, 2014). Nearly all such examples come from more rearranged plastomes, making it difficult to reconstruct whether the relocation was direct (implying the involvement of a translocase or site‐specific recombinase) or the indirect result of some combination of overlapping inversions and/or differential IR expansions/contractions.

Among nonvascular land plants, plastomic rearrangements are rare, with just a few examples of inversions and IR boundary shifts and no putative cases of transposition/translocation. Indeed, plastomes from most species are fully collinear, including duplications of four ribosomal RNAs (rRNAs) and five transfer RNAs (tRNAs) within the IR (Grosche *et al*., 2012; Bell *et al*., 2014; Villarreal *et al*., 2018), indicating that this structural arrangement was stably inherited from the common ancestor of all land plants (Mower & Vickrey, 2018). The rare exceptions of structural change involve a *c*. 70 kb inversion that characterizes mosses within the Funariidae (Sugiura *et al*., 2003; Goffinet *et al*., 2007), a 1 kb inversion unique to the parasitic liverwort *Aneura mirabilis* (Wickett *et al*., 2008), and a *c*. 7 kb IR expansion that is specific to hornworts in Anthocerotaceae (Villarreal *et al*., 2013).

By contrast, inversions and IR boundary shifts are recurrent themes in the evolution of euphyllophyte (i.e., angiosperm, gymnosperm, and fern) plastomes (Zhu *et al*., 2016; Mower & Vickrey, 2018). For example, a *c*. 35 kb inversion is shared by all species, suggesting that it occurred in the euphyllophyte ancestor (Raubeson & Jansen, 1992). Major expansions of the IR distinguish angiosperm and gymnosperm plastomes from all other land plant lineages, which were followed by further episodes of inversions and IR boundary shifts affecting one to many genes in particular descendant lineages (Wicke *et al*., 2011; Jansen & Ruhlman, 2012; Mower & Vickrey, 2018). Gene transpositions/translocations have been invoked for some highly rearranged plastomes (Milligan *et al*., 1989; Cosner *et al*., 1997, 2004; Chumley *et al*., 2006; Knox, 2014), but dense sampling of plastomes suggested that overlapping inversions provide an equally good or better explanation for most, but not necessarily all, examples (Lee *et al*., 2007; Cai *et al*., 2008; Haberle *et al*., 2008; Knox, 2014; Weng *et al*., 2014). Among ferns, a small number of plastomic inversions are shared by all living species (Gao *et al*., 2011; Grewe *et al*., 2013; Mower & Vickrey, 2018), while descendant lineages have experienced either multiple IR expansions from a plesiomorphic arrangement or multiple IR contractions following an early expansion (Grewe *et al*., 2013; Zhu *et al*., 2016; Mower & Vickrey, 2018). The relocation of several genes from the LSC to the IR of most leptosporangiate ferns was inferred to result from two overlapping inversions (Hasebe & Iwatsuki, 1992; Stein *et al*., 1992; Raubeson & Stein, 1995). Other structural changes among fern plastomes may have been facilitated by the presence of mobile elements, termed MORFFO, within the plastome (Robison *et al*., 2018).

Given that lycophytes are the sister lineage to all other extant vascular plants and comprise three distinctive lineages (clubmosses, spikemosses, and quillworts) that have each existed for >300 million years (Larsén & Rydin, 2016; Testo *et al*., 2018), it is perhaps unsurprising that their plastomes exhibit diverse characteristics. Plastomes from *Huperzia lucidula* and *Huperzia serrata* are nearly identical to one another (Guo Z. Y. *et al*., 2016), and, except for a small expansion of the IR, they are collinear with nonvascular plant plastomes, implying a plesiomorphic retention of gene order from the land plant ancestor (Wolf *et al*., 2005; Mower & Vickrey, 2018). By contrast, *Isoetes* and *Selaginella* harbor plastomes that are more rearranged (Tsuji *et al*., 2007; Smith, 2009; Karol *et al*., 2010; Xu *et al*., 2018). The plastome from *Isoetes flaccida* exhibits two diagnostic changes, including an inversion affecting *chlL* and *chlN* and a putative translocation of *ycf2* from the LSC to the SSC (Karol *et al*., 2010). Plastomes from three *Selaginella* species (*S. moellendorffii*,* S. tamariscina*, and *S. uncinata*) are more diverse due to multiple inversions, putative translocations, and gene losses (Tsuji *et al*., 2007; Smith, 2009; Xu *et al*., 2018). The IR has evolved differently in all three genera, exemplified by minor expansions in *Huperzia* that captured portions of *ndhF* and exon 2 of *rps12* (Wolf *et al*., 2005; Karol *et al*., 2010), by an independent and slightly larger IR expansion in *Isoetes* that assimilated *rps7* and exons 2 and 3 of *rps12* (Karol *et al*., 2010), and by distinct IR expansions in different *Selaginella* species accompanied by multiple losses of tRNA genes (Tsuji *et al*., 2007; Smith, 2009; Xu *et al*., 2018).

The variable nature of plastome sequences among lycophytes indicates that multiple evolutionary events must have occurred, but the limited availability of complete plastomes has hampered attempts to reliably reconstruct the series of events that produced these changes. To date, only six species from three genera have been sequenced from lycophytes, a group that comprises *c*. 1300 species and 18 genera (Schuettpelz *et al*., 2016). To improve plastome sampling among lycophytes, we generated complete plastome sequences from seven species (*Dendrolycopodium obscurum*,* Diphasiastrum digitatum*,* Isoetes malinverniana*,* Isoetes piedmontana*,* Lycopodium clavatum*,* Selaginella kraussiana*, and *Selaginella lepidophylla*) and resequenced the plastome of an eighth species (*H. lucidula*). These new sequences were used to assess the range of plastomic diversity among species and, from this information, the evolutionary history of plastome change in lycophytes was reconstructed. These analyses uncovered a pattern of evolutionary stasis among Lycopodiaceae genomes contrasted with major genomic upheaval in *Selaginella*, including a novel genomic arrangement in which the large rRNA‐containing repeat copies shifted from an inverted to a direct orientation. The origins of this novel arrangement and its potential effects on the rate of nucleotide substitution were evaluated.

## Materials and Methods

### Sample collection and DNA sequencing

Tissue was collected from six species of lycophytes: *De. obscurum*,* Di. digitatum*,* H. lucidula*,* I. malinverniana*,* I. piedmontana* and *L. clavatum*. Total genomic DNA was extracted using a simplified CTAB procedure (Doyle & Doyle, 1987) or the Qiagen DNeasy Plant mini kit and then sequenced on the Illumina HiSeq or NextSeq platforms. DNA for *S. lepidophylla* was extracted and then sequenced using the PacBio platform, as described previously (VanBuren *et al*., 2018). For *S. kraussiana*, Illumina sequencing data were obtained from the NCBI Sequence Read Archive (accession no. SRR2037123). Details of sample collection and genomic sequencing are listed in Supporting Information Table S1.

### Plastome assembly

The *S. lepidophylla* plastome was identified from contigs in the PacBio assembly produced by Canu v1.7 from the published nuclear genome project (VanBuren *et al*., 2018). To assemble plastomes for the remaining species, a full data set of all raw Illumina sequence reads and a reduced data set including a subset of 20 million raw reads (which often contains sufficient plastomic coverage for high‐quality assembly) were generated for each species. The full and reduced data sets were each independently assembled using Velvet v.1.2.10 (Zerbino & Birney, 2008) and Spades v.3.11.1 (Bankevich *et al*., 2012). For both assemblers, kmers were set dependent upon the sequenced read length: kmers 79, 95, 111 and 127 were used with 151 bp reads; kmers 69, 83, 97 and 111 were used with 125 bp reads; and kmers 61, 71, 81 and 91 were used with 101 bp reads. For Spades assemblies, the careful flag was activated and coverage cutoff was set either to 10 (for the full‐read data set) or 5 (for the 20‐million‐read data set). For Velvet assemblies, independent runs were executed without scaffolding using pairwise combinations of kmers and expected coverage values (set to 50, 100, 200, 500, and 1000), as previously described (Guo *et al*., 2014; Sigmon *et al*., 2017).

For each of the draft assemblies produced from the two data sets and two assemblers, plastid contigs were identified by using Blastn searches with *H. lucidula* (GenBank accession no. AY660566), *I. flaccida* (GU191333) and *S. moellendorffii* (HM173080) plastome sequences as queries. For each species, a minimum of three of the best draft assemblies (regardless of assembler or data set used) was identified based on maximal average length of identified plastid contigs. These best assemblies were manually aligned, from which a final consensus sequence was generated.

Polymerase chain reaction (PCR) and Sanger sequencing were used to verify the sequence and structure of the plastome at several notable positions. The direct orientation of the large repeat in *S. kraussiana* was verified by sequencing PCR amplicons that spanned each boundary of the SC and repeat regions (Fig. S1). In addition, the plastomes of *De. obscurum*,* Di. digitatum*,* H. lucidula* and *L. clavatum* contain several repetitive regions composed of tandem repeats, with unit sizes ranging from one to several hundred nucleotides. The size and complexity of these repetitive regions were verified using primers anchored in nonrepetitive flanking regions. PCR reactions included an initial denaturation step for 3 min at 94°C, followed by 35 cycles of denaturation (94°C for 30 s), annealing (50–54°C for 30 s), and elongation (72°C for 90–150 s), and concluding with a final elongation step for 10 min at 72°C. PCR products were sequenced on both strands by GenScript (Piscataway, NJ, USA).

### Plastome annotation

The *I. piedmontana* plastome was initially annotated using the Live Annotation feature in Geneious v.10.2.2 (Kearse *et al*., 2012), with the annotation of *I. flaccida* (GU191333) as a template. All other plastomes were initially annotated using the online implementation of GeSeq (Tillich *et al*., 2017), with annotated plastomes from *H. lucidula* (AY660566), *H. serrata* (KX426071), *I. flaccida* (GU191333), *S. moellendorffii* (HM173080) and *S. uncinata* (AB197035) selected as references. Annotation of tRNAs was performed using Aragorn (using the plant chloroplast genetic code) and tRnascan‐se (set for organellar tRNAs) as implemented by GeSeq.

To ensure that all protein‐coding genes, tRNAs, rRNAs, introns and splicing junctions were detected and properly annotated in all lycophyte plastomes evaluated in this study (including all eight newly generated plastomes and the six plastomes obtained from GenBank), a Blastn search was conducted against each plastome using a query set of all plastid genes (including intron sequences when present) collected from a diversity of land plant plastomes. In addition, for protein‐coding genes, tBlastn searches were conducted for increased sensitivity. These Blast validations identified several exons and introns that were often missed by GeSeq, involving the short first exons in *psbB*,* psbD*, and *rpl16* and the *trans*‐spliced *rps12* gene, as well as several genes and introns that were missing or incorrectly annotated in some plastomes in GenBank. These annotation errors were corrected before comparative analyses. Finally, to assess whether MORFFO‐like mobile elements were present in lycophyte plastomes, Blastn and tBlastn searches were also conducted using intact *morffo* gene sequences as queries. All newly sequenced plastomes were deposited in GenBank under accession nos. MH549637–MH549643 and MK089531.

### Comparative genomic analyses

All comparative analyses were performed using the corrected annotations for all 14 examined lycophyte plastomes. Therefore, any variation among genes and introns is likely to reflect true variation in genomic content rather than annotation errors. The presence of genes and introns was taken from the corrected annotations, all of which were verified by the Blastn and tBlastn searches performed during intron annotation. The absence of genes was confirmed by an absence of any hits in the Blastn and tBlastn searches. Pseudogenes were scored when reading frames had frameshifts and/or substantial truncations. Additionally, because no U‐to‐C editing has been detected in *Selaginella* (Hecht *et al*., 2011; Oldenkott *et al*., 2014), the presence of premature stop codons was used as another indicator of pseudogenization in *Selaginella* plastomes. Collinearity among plastomes was assessed by comparison of linear genome maps generated by Ogdraw (Lohse *et al*., 2007). Ancestral reconstruction of inversion events and gene and intron losses was performed manually using the maximum parsimony criterion, which minimizes the number of inferred changes in a phylogenetic context.

### Substitution rate calculations

Protein‐coding and rRNA genes were collected from the plastomes of 12 different species of lycophytes and a diverse sampling of seven other land plants: the angiosperm *Magnolia tripetala* (KJ408574), the gymnosperm *Ginkgo biloba* (KP099648), the ferns *Angiopteris angustifolia* (KP099647) and *Ophioglossum californicum* (KC117178), the hornwort *Anthoceros formosae* (AB086179), the moss *Physcomitrella patens* (KY126308), and the liverwort *Marchantia polymorpha* (LC192146). Individual protein genes were aligned by codon with TranslatorX v.1.1 (Abascal *et al*., 2010) using default settings, while individual rRNA genes were aligned with Muscle v.3.8.31 (Edgar, 2004) using default settings. Three separate protein‐gene data sets, containing either the 51 protein genes that are SC in all lycophytes, the three protein genes (*ndhB*,* psbM*,* rps7*) that are duplicated in *S. kraussiana*, or the *rps4* gene that is duplicated in *S. moellendorffii* and *S. tamariscina*, were created with Gblocks v0.91b (Castresana, 2000) in codon mode with gaps allowed in up to half of sequences (t = c b5 = h a = y). An rRNA data set containing all four rRNA genes was generated with Gblocks in DNA mode with gaps allowed in up to half of sequences (t = d b5 = h a = y). All four trimmed data sets are provided in Dataset S1.

A maximum‐likelihood tree was generated from the 51‐gene data set with Phyml v.3.0 (Guindon *et al*., 2010) using a GTR + G + I nucleotide model, which was determined as the best‐fitting model by the ModelTest (Posada & Crandall, 1998) implementation in Hyphy v.2.2.4 (Pond *et al*., 2005), and all model parameters were estimated during the Phyml run. The recovered topology fully agreed with recent phylogenetic analyses of lycophytes (Field *et al*., 2016; Larsén & Rydin, 2016; Zhou *et al*., 2016). Sequence divergence was calculated with Hyphy for each branch in a tree that was constrained according to the maximum‐likelihood topology from the phylogenetic analysis of 51 SC genes. Branch lengths representing synonymous sequence divergence (*d*_S_) were estimated from each of the protein‐gene data sets using a local MG94CustomF3x4 codon model. The best‐fitting codon model was determined by the CodonModelCompare analysis in Hyphy to be 012232 for the 51‐gene data set, 012312 for the *ndhB *+ *psbM *+ *rps7* data set, and 012012 for the *rps4* data set. Branch lengths representing total rRNA sequence divergence (*d*) were estimated from the concatenated rRNA data set using a GTR + G + I nucleotide model, which was determined to be the best fit by the Hyphy implementation of ModelTest.

## Results

### Lineage‐specific variation in plastome size and GC content among lycophytes

Plastome size and GC content among the three major lineages of lycophytes vary in a lineage‐specific manner (Table 1). Plastomes from Lycopodiaceae are largest (152–161 kb) with the lowest GC content (35–36%), *Isoetes* plastomes are intermediate in size (145–146 kb) and GC content (38%), and *Selaginella* plastomes are smallest (115–144 kb) and the most GC rich (51–55%). The size of the LSC correlates directly with overall genome size, being largest in Lycopodiaceae and smallest in *Selaginella*, whereas SSC size exhibits an inverse trend. The two‐fold variation in size of the large plastid repeat, at 7.3–18 kb, is driven primarily by expansions, contractions, gene losses, and inversions (described in Results section). GC content within the IR is consistently higher than the SC regions in all lycophytes, due at least in part to the elevated GC content of rRNA genes.

**Table 1:** General characteristics of lycophyte plastomes

	Lycopodiaceae						Isoetaceae			Selaginellaceae				
	Dobs	Ddig	Lcla	Hluc1	Hluc2	Hser	Ifla	Imal	Ipie	Skra	Slep	Smoe	Stam	Sunc
Length (bp)	160 877	159 614	151 819	154 373	154 368	154 176	145 303	145 535	145 030	129 971	114 693	143 775	126 399	144 170
Repeat (bp)	17 742	16 885	12 417	15 314	15 314	15 313	13 118	13 217	13 042	14 597	7308	12 114	12 831	12 789
LSC (bp)	105 928	106 400	105 643	104 088	104 083	103 892	918 62	91 715	91 748	54 728	80 625	83 665	53 170	77 706
SSC (bp)	19 465	19 444	21 342	19 657	19 657	19 658	27 205	27 386	27 198	46 049	19 452	35 882	47 567	40 886
GC content (%)	35.0	35.7	34.5	36.2	36.3	36.3	37.9	38.0	38.0	52.3	51.9	51.0	54.0	54.8
Repeat GC (%)	42.3	43.4	46.6	44.9	44.9	44.9	48.0	47.9	48.0	56.5	57.1	55.7	55.2	57.5
LSC GC (%)	33.1	33.8	32.3	34.4	34.4	34.4	36.4	36.5	36.5	50.9	51.2	49.9	53.4	54.3
SSC GC (%)	31.9	32.9	31.3	32.8	32.8	32.8	33.4	33.3	32.7	51.5	51.3	50.5	54.0	54.3
Genes	122	122	122	122	122	122	118	118	118	85	80	93	68	93
Protein	87	87	87	87	87	87	82	82	82	71	64	76	58	77
rRNA	4	4	4	4	4	4	4	4	4	4	4	4	4	4
tRNA	31	31	31	31	31	31	32	32	32	10	12	13	6	12
Introns	22	22	22	22	22	22	21	21	21	9	7	11	7	11

LSC, large single copy; SSC, small single copy; Dobs, *Dendrolycopodium obscurum*; Ddig, *Diphasiastrum digitatum*; Lcla, *Lycopodium clavatum*; Hluc1 and Hluc2, *Huperzia lucidula*; Hser, *Huperzia serrata*; Ifla, *Isoetes flaccida*; Imal, *Isoetes malinverniana*; Ipie, *Isoetes piedmontana*; Skra, *Selaginella kraussiana*; Slep, *Selaginella lepidophylla*; Smoe, *Selaginella moellendorffii*; Stam, *Selaginella tamariscina*; Sunc, *Selaginella uncinata*.

### Extensive variation in gene and intron content among lycophyte plastomes

**Figure 1:**
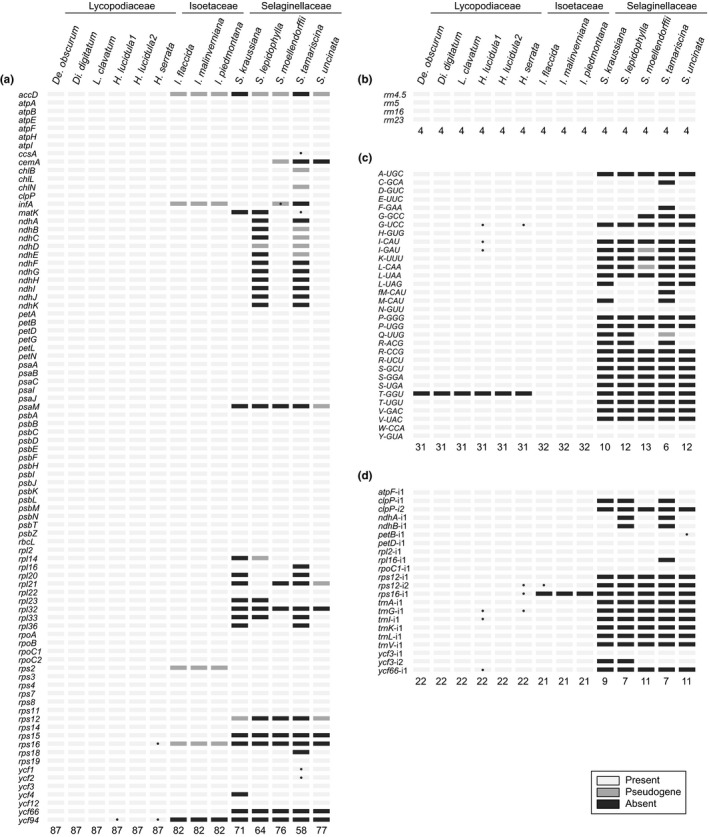
Functional content of lycophyte plastomes. (a) Protein‐coding genes. (b) rRNA genes. (c) tRNA genes. (d) Introns. Total counts are listed at the bottom of each functional category. Annotations for specific genes and introns that were corrected in this study are marked with a dark gray circle.

The repertoire of genes and introns varies widely among lycophyte plastomes, which again follows a lineage‐specific trend (Table 1). For protein‐coding genes (Fig. 1a), all six sequenced Lycopodiaceae plastomes contain the same set of 87 intact (and presumably functional) protein‐coding genes, a count that includes the recently described open reading frame (ORF) named *ycf94* (Song *et al*., 2018). Additionally, a frameshift within the *ycf2* gene was characteristic of Lycopodioideae (*De. obscurum*,* Di. digitatum*, and *L. clavatum*), suggesting that this gene may now operate as two separate reading frames (hereafter referred to as *ycf2n* and *ycf2c*). The three *Isoetes* species have a slightly reduced repertoire of 82 intact protein genes in their plastomes. Of the five lost genes, nonfunctional remnants remain for *accD*,* infA*,* rps2* and *rps16* but no fragment of *ycf94* was detected in any *Isoetes* plastome.

The five species of *Selaginella* contain many fewer intact protein‐coding genes, indicating substantial gene loss (Fig. 1a). Gene loss is most pronounced in the recently published plastome from *S. tamariscina* and the newly elucidated *S. lepidophylla* plastome, with only 58 and 64 intact protein genes remaining, respectively. Notably, both of these plastomes lack functional copies of the *ndh* complex (Fig. 1a; Xu *et al*., 2018), similar to observations in a variety of other land plants (Wakasugi *et al*., 1994; Wickett *et al*., 2008; Blazier *et al*., 2011), while the *S. kraussiana* and *S. lepidophylla* plastomes lack the maturase gene *matK*, which encodes an intron splicing factor (Vogel *et al*., 1997; Zoschke *et al*., 2010). All *Selaginella* plastomes have also lost numerous ribosomal protein genes, as well as a variety of other genes, that are universally present in all other lycophytes. The status of *infA* is uncertain in some *Selaginella* species; an intact but highly divergent copy exists in *S. uncinata*, which was tentatively scored as functional in this study, whereas it has probably become a pseudogene in *S. moellendorffii* (due to the presence of two internal stop codons which cannot be eliminated because U‐to‐C RNA editing is absent from *S. moellendorffii*). In ferns, particular MORFFO mobile elements have led to structural rearrangements (Robison *et al*., 2018); however, no homology to any *morffo*‐like genes was detected in any of the 14 lycophyte plastomes.

Among RNA genes, all lycophytes have retained the standard set of four ribosomal RNA (rRNA) genes (Fig. 1b), whereas transfer RNA (tRNA) content is more variable among lineages (Fig. 1c). All three *Isoetes* plastomes have retained 32 tRNA genes, including a *trnT‐GGU* gene that is common throughout land plants (Grosche *et al*., 2012; Grewe *et al*., 2013; Bell *et al*., 2014; Guo *et al*., 2014; Villarreal *et al*., 2018). Plastomes from Lycopodiaceae have retained nearly the same set of tRNAs, except for the loss of *trnT‐GGU*. By contrast, all five species of *Selaginella* have lost the majority of their plastid tRNAs. This loss is most pronounced in *S. tamariscina* and *S. kraussiana*, which harbor only 6 and 10 detectable tRNAs in their plastomes, respectively.

Intron content also varies widely among lycophytes (Fig. 1d). All six Lycopodiaceae species contain a complete set of 22 plastid introns that are widely present across land plants. *Isoetes* plastomes lack just one of these introns, and its absence can be attributed to the loss of function and subsequent degradation of the host *rps16* gene. By contrast, 16 different introns have been variously lost from one or more *Selaginella* species, most extensively from *S. tamariscina* and *S. lepidophylla* plastomes. The absence of 13 introns (from *ndhA*,* ndhB*,* rpl16*,* rps12*,* rps16*,* ycf66*, and six tRNAs) can be attributed to loss of their host genes. For the remaining three intron losses (from *clpP* and *ycf3*), the host plastid genes are still present and presumably functional.

### Plesiomorphic retention of plastome structure in Lycopodiaceae

**Figure 2:**
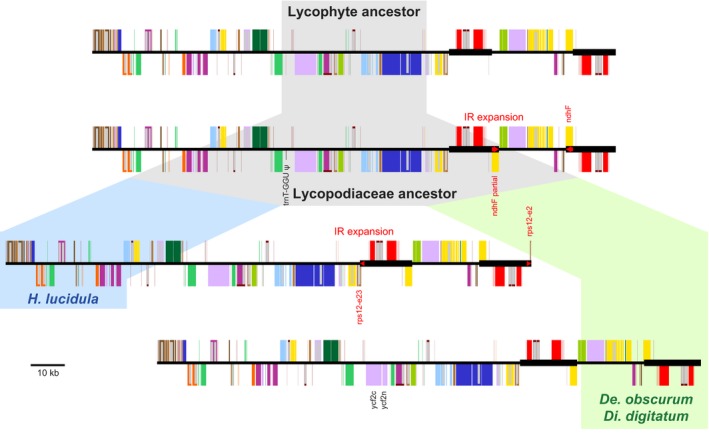
Structural evolution of Lycopodiaceae plastomes. The loss of *trnT‐GGU* is marked with a ψ. Inverted repeat (IR) expansions are denoted with red arrows, and genes affected by the IR expansion are listed in red text. The two genes arising from the split of *ycf2* are labeled.

Plastome structure and gene order are highly conserved among the Lycopodiaceae species examined in this study (Fig. 2). All six plastomes shared the loss of *trnT‐GGU* and all but *L. clavatum* had an expanded IR that included nearly all of the *ndhF* gene. Based on current sampling, these two evolutionary events can be most parsimoniously inferred to have occurred in the common ancestor of Lycopodiaceae. In *L. clavatum*, however, only about 200 bp of the *ndhF* gene were located within the IR, suggesting that this genome experienced a more recent IR contraction. Both *Huperzia* species shared another minor IR expansion that moved exon 2 and part of intron 2 of *rps12* into the IR, whereas the split of *ycf2* into *ycf2n* and *ycf2c* was specific to the Lycopodioideae (*De. obscurum*,* Di. digitatum*, and *L. clavatum*). Other than these minor changes, plastome gene order in Lycopodiaceae is collinear with plastomes from nonvascular land plants, and this conserved synteny enabled a robust reconstruction of the ancestral lycophyte plastome (Fig. 2, top).

### Multiple shifts between inverted and direct orientations of the large plastid repeat in *Selaginella*

**Figure 3:**
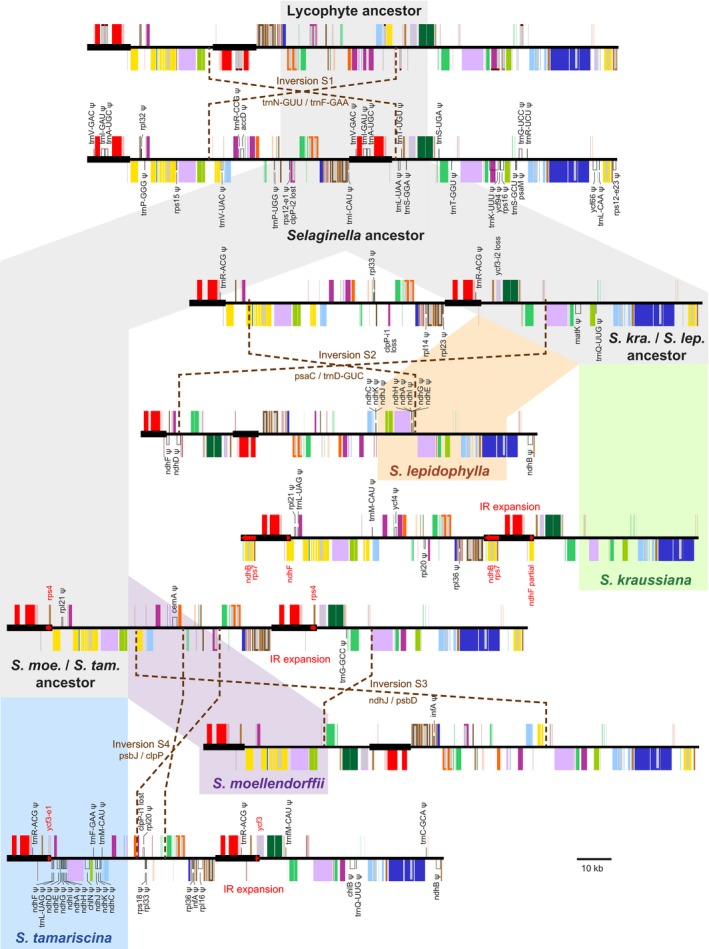
Structural evolution of *Selaginella* plastomes. Pseudogenes and lost genes are marked with a ψ and listed in black text. Inversion endpoints are marked by brown dotted lines, and the genes closest to each endpoint are listed in brown text. Inverted repeat (IR) expansions are denoted with red arrows, and genes affected by the IR expansion are listed in red text.

By contrast with the evolutionary stasis of Lycopodiaceae plastomes, major structural changes have reorganized *Selaginella* plastomes (Fig. 3). In the *S. kraussiana* and *S. tamariscina* plastomes, the most conspicuous change is the relative orientation of the large rRNA‐containing repeat copies, which are arranged as a pair of direct repeats (DR) instead of the canonical IR orientation observed in other land plant plastomes. Comparison with the ancestral lycophyte plastome demonstrated that this unique IR‐to‐DR transition occurred by a single inversion event that fully spanned the IR (Inversion S1 in Fig. 3), with one endpoint in the ancestral LSC (between *trnF‐GAA* and *rps4*) and the other in the ancestral SSC (just beyond the IR/SSC boundary). The direct orientation of the *S. kraussiana* repeat was independently confirmed by PCR and Sanger sequencing across all four DR/SC junctions (Fig. S1), while the DR structure for *S. tamariscina* was confirmed in another study (Xu *et al*., 2018).

Evolutionary reconstruction of the plastomes of *S. moellendorffii*,* S. lepidophylla* and *S. uncinata* revealed that this same Inversion S1 also affected these plastomes, demonstrating that this event occurred in the common ancestor of all five *Selaginella* species (Figs 3, S2). In the *S. lepidophylla* and *S. moellendorffii* plastomes, Inversion S1 was followed by a second inversion (Inversion S2 for *S. lepidophylla* and Inversion S3 for *S. moellendorffii*) that also fully spanned the rRNA‐containing repeat, which independently reverted the DR back to a canonical IR orientation in the two species (Fig. 3). In the *S. uncinata* plastome, Inversion S1 was followed by at least four additional inversions and a tandem duplication of the *trnQ* and *psbK* genes, which collectively accounted for all of the various relocations and rearrangements in this plastome relative to the lycophyte ancestor (Fig. S2). Three of these *S. uncinata* inversions also spanned the rRNA repeat (Inversions S5, S7 and S8 in Fig. S2), which sequentially caused the orientation to revert from a DR to an IR, then invert back to a DR, and then revert once again to an IR. Additional plastomic inversions in *S. tamariscina* (Inversion S4 in Fig. 3) and *S. uncinata* (Inversion S6 in Fig. S2) did not affect the orientation of the repeat but instead inverted gene order in other regions of the plastome.

In addition to inversion events, these ancestral plastome reconstructions allowed inference of the relative timing of IR expansions as well as gene and intron losses among *Selaginella* species (Figs 3, S2). Many genes and introns were missing from all sampled *Selaginella* species, so their loss can be most parsimoniously interpreted as having occurred in the common ancestor of the five species, while other gene and intron losses occurred in particular descendant lineages. Most IR expansions were inferred to have occurred independently in the five species, except that the IR expansion that captured *rps4* probably occurred in the ancestor of *S. moellendorffii*¸ *S. tamariscina* and *S. uncinata*.

### Additional shifts in repeat orientation in *Isoetes* plastomes

**Figure 4:**
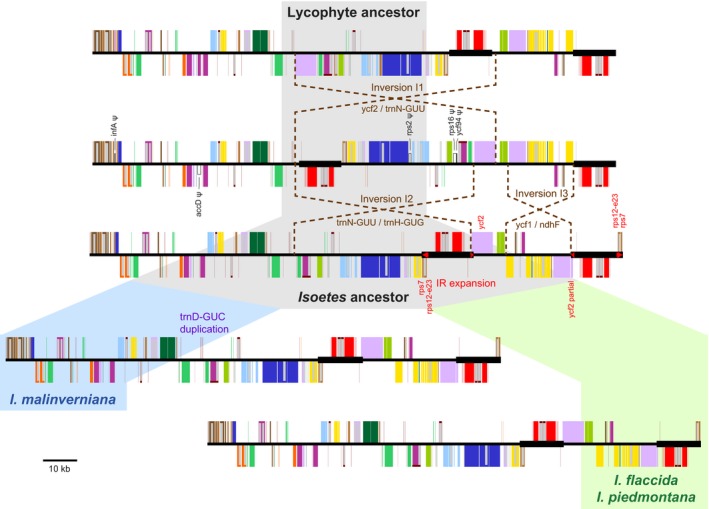
Structural evolution of *Isoetes* plastomes. Pseudogenes and lost genes are marked with a ψ and listed in black text. Inversion endpoints are marked by brown dotted lines, and the genes closest to each endpoint are listed in brown text. Inverted repeat (IR) expansions are denoted with red arrows, and genes affected by the IR expansion are listed in red text.

Compared with the ancestral lycophyte plastome, the main distinguishing features of the three sequenced *Isoetes* plastomes were the relocation of *ycf2* from the LSC into the SSC, an inversion within the SSC, and a minor IR expansion (Fig. 4). The relocation of *ycf2* can be attributed to two overlapping inversions (Inversion I1 and I2 in Fig. 4). Inversion I1 extended from *ycf2* to just beyond the IR/SSC boundary, while Inversion I2 endpoints involved the same region except it did not include *ycf2*, which effectively stranded *ycf2* in the SSC. Importantly, both of these inversion events in the *Isoetes* plastome fully spanned the IR region. Therefore, as also detected for *Selaginella*, the *Isoetes* lineage is inferred to have experienced a period in which the IR existed in a DR orientation, with a subsequent reversion back to an IR orientation. A third inversion (Inversion I3 in Fig. 4) was localized within the SSC that inverted most genes (from *ycf1* to *ndhF*) relative to the *chlN*–*chlL*–*ycf2* gene cluster. A small IR expansion resulted in the capture of a small part of *ycf2* from the SSC and exons 2 and 3 of *rps12* and *rps7* from the LSC. Finally, *trnD‐GUC* was tandemly duplicated specifically in *I. malinverniana* (Fig. 4).

### Substitution rates for genes in single‐copy and repeat regions in *Selaginella* plastomes

**Figure 5:**
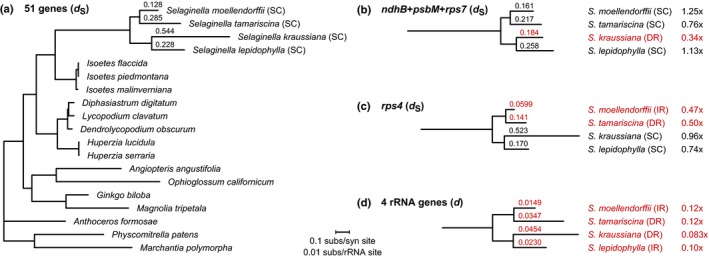
Substitution rate variation among lycophyte species and genes. (a) Synonymous divergence (*d*_S_) tree estimated from 51 concatenated genes that are single copy (SC) in all lycophytes. (b) *d*_S_ tree estimated from concatenated *ndhB *+ *psbM *+ *rps7* genes that are duplicated in *Selaginella kraussiana*. (c) *d*_S_ tree estimated from *rps4*, which is duplicated in *S. moellendorffii* and *S. tamariscina*. (d) Overall divergence (*d*) tree estimated from four rRNA genes that are duplicated in all *Selaginella* species. Species with duplicated genes are shown in red. Terminal branch lengths for all *Selaginella* species are listed, and a ratio of branch lengths is given for each species in (b–d) relative to branch lengths in (a). All trees are drawn to the scale shown at bottom. IR, inverted repeat; DR, direct repeat.

The IR exhibited reduced substitution rates in plant plastomes due to a copy‐dependent repair mechanism driven by homologous recombination, but it is unknown whether repeats in a DR orientation can promote homologous recombination or copy‐dependent repair. To explore this issue, we first established the relative substitution rate for SC genes by calculating synonymous sequence divergence (*d*_S_) of 51 genes that are SC in all lycophyte plastomes (Fig. 5a). Overall, SC *d*_S_ is substantially higher for *Selaginella* compared with other lycophytes. After the split between *Isoetes* and *Selaginella*, SC *d*_S_ is 5–7 times higher in *Selaginella* relative to *Isoetes*, with the highest SC *d*_S_ in *S. kraussiana*, which is consistent with previous studies reporting elevated sequence divergence in *Selaginella* (Korall & Kenrick, 2004; Zhou *et al*., 2016).

To test whether genes within the DR have reduced substitution rates relative to SC genes, SC *d*_S_ branch lengths were compared with corresponding *d*_S_ branches for the concatenated *ndhB *+ *psbM *+ *rps7* genes (which are duplicated in the DR of *S. kraussiana*) and for *rps4* (which is duplicated in the DR of *S. tamariscina*). For the concatenated *ndhB *+ *psbM *+ *rps7* genes (Fig. 5b), the terminal *S. kraussiana* branch (representing DR *d*_S_) was one‐third the length of the corresponding SC *d*_S_ branch for *S. kraussiana* from the 51‐SC‐gene tree (0.184 vs 0.544 substitutions/site). Similarly, in the *rps4* tree (Fig. 5c), the terminal *S. tamariscina* branch (representing DR *d*_S_) was less than one‐half the length of the corresponding SC *d*_S_ branch from the 51‐SC‐gene tree (0.141 vs 0.285). These results are consistent with a reduced substitution rate for DR genes in *S. kraussiana* and *S. tamariscina*. As a contrast, *d*_S_ branch lengths were roughly equivalent between the *ndhB*–*psbM*–*rps7* tree and the 51‐SC‐gene tree for *S. moellendorffii* (0.161 vs 0.128), *S. tamariscina* (0.217 vs 0.285), and *S. lepidophylla* (0. 258 vs 0.228), consistent with the SC status of *ndhB*–*psbM*–*rps7* in these three species. Likewise, *d*_S_ branch lengths were roughly equivalent in the trees from *rps4* (which is SC in *S. kraussiana* and *S. lepidophylla*) and the 51 SC genes for *S. kraussiana* (0.523 vs 0.544) and *S. lepidophylla* (0.170 vs 0.228).

As an additional test, SC *d*_S_ from the 51‐gene tree was compared with overall sequence divergence (*d*) from a tree constructed from all four rRNA genes (Fig. 5d). Given that rRNA genes are duplicated in all *Selaginella* plastomes, and under the assumption that effects of selection on rRNA genes are roughly equivalent for each species, we would expect rRNA *d* to correlate with SC *d*_S_ in an equivalent manner for all species if the DR experiences copy‐correction activity. Indeed, the rRNA *d* was 8–12 times lower than SC *d*_S_ for all *Selaginella* species, indicating that they have experienced similar reductions in rRNA substitution rate relative to SC rate despite differences in repeat orientation among species.

## Discussion

### Evolutionary stasis in Lycopodiaceae plastomes contrasts with exceptional evolutionary eccentricities in *Selaginella* plastomes

Comparative analysis of 14 plastomes (eight newly generated for this study) from species spanning the breadth of extant lycophytes has revealed substantial variation in size, content, structure and substitution rate, driven primarily by evolutionary eccentricities in *Selaginella* plastomes. The 115–161 kb range of lycophyte plastome sizes, due mostly to variation among *Selaginella* plastomes (Table 1), closely aligns with the typical 120–160 kb range observed for most other photoautotrophic vascular plants (Wicke *et al*., 2011; Jansen & Ruhlman, 2012; Mower & Vickrey, 2018). The 51–55% GC content among the five *Selaginella* plastomes (Table 1) is substantially higher than for all other land plant plastomes sequenced to date, as previously noted for *S. moellendorffii* and *S. uncinata* (Tsuji *et al*., 2007; Smith, 2009), while the plastomic GC content of 35–38% for Lycopodiaceae and *Isoetes* (Table 1) is unremarkable compared with other vascular plants (Jansen & Ruhlman, 2012; Grewe *et al*., 2013; Chaw *et al*., 2018). Similarly, substitution rates are substantially higher in *Selaginella* plastid genes compared with other lycophytes and a representative sampling of other land plants (Fig. 5). Another unique characteristic of the *Selaginella* plastome is an extreme level of cytidine‐to‐uracil (C‐to‐U) RNA editing, with over 3400 sites for *S. uncinata*, the highest of any land plant, whereas RNA editing in other lycophytes was predicted to be much lower, at 340 sites in *H. lucidula* and 460 sites in *I. flaccida* (Oldenkott *et al*., 2014).

With respect to plastid gene and intron content, *Selaginella* is again an extreme outlier due to unprecedented gene and intron losses, which contrasts sharply with the minimal losses from Lycopodiaceae and *Isoetes* (Table 1; Fig. 1). The total unique repertoire (i.e. not counting duplicates in the IR) of 68–93 genes and 7–11 introns in *Selaginella* plastomes is the lowest among all photoautotrophic land plants, which typically harbor 110–120 genes and 18–22 introns in their plastomes (e.g. Grosche *et al*., 2012; Jansen & Ruhlman, 2012; Grewe *et al*., 2013; Bell *et al*., 2014; Villarreal *et al*., 2018). This major reduction in plastid gene content was driven primarily by the loss of 60–80% of the 32 tRNAs as well as many ribosomal protein genes, particularly from *S. tamariscina* and *S. kraussiana* plastomes, and all *ndh* genes from the *S. tamariscina* and *S. lepidophylla* plastomes. Homologs to some, but not all, of the lost plastid genes were identified in the nuclear genomes of *S. tamariscina* and *S. moellendorffii*, indicating functional intracellular transfer of these genes to the nuclear genome (Smith, 2009; Xu *et al*., 2018). The lack of detection of nuclear homologs for the other lost plastid genes may indicate a complete loss of function from the organism, as observed previously for *ndh* genes in many seed plants (Ruhlman *et al*., 2015), or an inability to detect nuclear homologs for generally short‐ and fast‐evolving genes (Smith, 2009). Gene losses from *Selaginella* and *Isoetes* plastomes were the primary cause for most of the intron losses, whereas intron losses from functional *ycf3* and *clpP* genes suggested a role for retroprocessing, which is a process involving the reverse transcription and genomic reintegration of a spliced transcript. Retroprocessing has also been postulated as the primary mechanism of intron loss from plant mitochondrial genomes (Cuenca *et al*., 2016; Grewe *et al*., 2016; Guo W. *et al*., 2016).

Plastome structure also varies substantially among lycophytes. The *S. kraussiana* and *S. tamariscina* plastomes stand alone among land plants due to their unique DR orientation (Fig. 3). The only comparable plastomes with DR orientations are found among various red algae in the Bangiophyceae and Florideophyceae (Lee *et al*., 2016). The *S. uncinata* plastome is the most rearranged among lycophytes, with at least five inversions and a tandem duplication required to reconcile its plastome structure (Fig. S2). By contrast, Lycopodiaceae plastomes are unique among vascular plants for their similarity to nonvascular plant plastomes; indeed, the retention of most plastid genes and introns and conserved synteny compared with nonvascular land plants indicated that plastome structure has remained largely unchanged in Lycopodiaceae and most nonvascular land plants since the last common ancestor of all land plants *c*. 500 million years ago (Magallon *et al*., 2013; Morris *et al*., 2018).

Collectively, these results show that *Selaginella* plastomes exhibit a wide variety of atypical features compared with all other land plants, whereas Lycopodiaceae plastomes are remarkable for their lack of change. The confinement of these extreme evolutionary eccentricities to *Selaginella* plastomes indicates that they probably arose within the *Selaginella* lineage. The extent of these genomic changes varies among the five sampled *Selaginella* species. The DR plastomes of *S. kraussiana* and *S. tamariscina* tend to have smaller sizes (Table 1), fewer genes and introns (Table 1; Fig. 1), and the fastest substitution rates (Fig. 5), although the *S. lepidophylla* plastome with an IR structure is even smaller and has lost many genes as well. Denser sampling of *Selaginella* plastomes is needed to more fully assess the evolutionary dynamics of these genomic features within the genus, and to evaluate whether a DR structure promotes other types of structural change.

### Broadly correlated evolutionary dynamics of lycophyte plastomes and mitochondrial genomes

Intriguingly, the *Selaginella* mitochondrial genome exhibits many of the same extreme evolutionary trends as the plastome. This includes a small genome with a high GC content, in fact the highest among eukaryotes (Hecht *et al*., 2011). Mitochondrial gene content is also substantially lower for *Selaginella* relative to *Isoetes* and *Phlegmariurus* (Grewe *et al*., 2009; Hecht *et al*., 2011; Liu *et al*., 2012). Mitochondrial gene loss was primarily due to loss of tRNAs and ribosomal protein genes (Hecht *et al*., 2011), paralleling the predominant pattern of gene loss from the plastome. Furthermore, analyses of a few mitochondrial genes and introns indicated that the mitochondrial substitution rate is substantially higher for *Selaginella* relative to other lycophytes (Wikstrom & Pryer, 2005; Guo & Mower, 2013), while the *Selaginella* mitochondrial transcriptome has the highest amount of C‐to‐U RNA editing relative to all other land plants (Hecht *et al*., 2011). The *Selaginella* mitochondrial genome is also highly recombinogenic (Hecht *et al*., 2011), while the *Phlegmariurus* genome is the least rearranged among vascular plants (Liu *et al*., 2012). However, one major inconsistency between the two organellar genomes relates to introns: mitochondrial intron content for all three sequenced lycophyte genomes is very high compared with other land plants (Grewe *et al*., 2009; Hecht *et al*., 2011; Liu *et al*., 2012), with more mitochondrial introns in *Selaginella* than in any other land plant, which is opposite to the pattern of massive intron loss from *Selaginella* plastomes.

A correlation between GC content and RNA editing was previously noted (Malek *et al*., 1996; Tsuji *et al*., 2007; Smith, 2009; Hecht *et al*., 2011), although it is unclear which is the causal factor or if both phenomena are the effect of some other driving force. For example, a GC bias for the increased rate of nucleotide substitution in *Selaginella* could potentially drive up both GC content and the number of edited sites in *Selaginella*. A higher mutation‐rate environment in the organellar genomes may have also facilitated the transfer of genes to the nuclear genome, although the extreme level of editing would likely require that these genes be transferred as RNA or as a DNA transfer of a retroprocessed gene. Correlated evolution of mitochondrial and plastid features could also be coordinated by the effect of one or more of the many nuclear factors involved in genomic maintenance and transcriptional processing that are shared between the two organelles (Delannoy *et al*., 2007; Takenaka *et al*., 2012; Gualberto & Newton, 2017). Ultimately, many more lycophyte organellar genomes are needed to assess the true degree of correlation of these features between the mitochondrial and plastid genomes, and the putative underlying causes of these correlations.

### DR orientation and implications for recombination and repair

The direct orientation of the rRNA‐containing repeat in some *Selaginella* plastomes has intriguing implications with respect to the activities of recombination and repair. In plastomes with an IR, it is now well established that the repeat copies undergo frequent homologous recombination, resulting in isomeric forms of the plastome that differ in the relative orientation of the LSC and SSC (Bohnert & Loffelhardt, 1982; Palmer, 1983). This recombinational activity is also known to promote copy‐dependent repair via gene conversion, reducing the substitution rate of genes that reside in the IR relative to those in the SC regions (Wolfe *et al*., 1987; Perry & Wolfe, 2002; Zhu *et al*., 2016). However, due to the novelty of plastomes with a DR arrangement, no studies have yet assessed whether homologous recombination occurs or promotes copy‐dependent repair in DR plastomes.

Our study demonstrates that genes within the DR of *S. kraussiana* and *S. tamariscina* have reduced substitution rates relative to SC genes (Fig. 5), consistent with the continued activity of copy‐dependent repair despite a DR arrangement. This finding implies that recombination is indeed occurring between the two repeat copies in these DR plastomes. Assuming a circular chromosome, recombination between the DR copies would result in a pair of subgenomic circles, each containing either the LSC or SSC and one copy of the plastomic repeat. However, many studies have raised questions about the circular chromosome model for plastomes, suggesting instead that the major form of the plastome is linear (e.g., Bendich, 2004; Oldenburg & Bendich, 2016). If the *Selaginella* plastome exists predominantly as linear molecules, then frequent recombination between repeats would generate a diverse population of linear molecules in which the repeat might separate the LSC and SSC, or two copies of the LSC, or two copies of the SSC. Studies using electron microscopy and electrophoretic separation are needed to assess the *in vivo* structure of the plastome in *Selaginella*.

### Overlapping inversions as the mechanism for gene relocation in lycophytes

Finally, our results demonstrate that there is no need to invoke direct transposition or intragenomic translocation, as mediated by a translocase or recombinase, for the relocation of plastid genes from the LSC to the SSC in *S. uncinata* and *S. moellendorffii*. Instead, overlapping inversions provide a more likely explanation, particularly because the intermediate step was captured in the plastomes from *S. kraussiana* and *S. tamariscina*, which produced the DR orientation in these two genomes (Fig. 3). The retention of this intermediate inversion step in the *S. kraussiana* and *S. tamariscina* plastomes provides strong evidence that inversion S1 occurred in the common ancestor of all five sampled *Selaginella* species, before any subsequent overlapping inversions. Subsequent inversion events, which broadly overlapped inversion S1, must then be inferred for *S. lepidophylla*,* S. moellendorffii* and *S. uncinata* to explain the reversion of the IR orientation. Importantly, the endpoints of the subsequent inversions were different in each of these plastomes, providing a clear explanation for the slightly different suite of genes that were ultimately relocated between the LSC and SSC of these species. Denser sampling of the many species in the clades represented by *S. lepidophylla*,* S. moellendorffii* and *S. uncinata* should further pinpoint the relative timing of these subsequent inversions, and may reveal species with plastomes that are even more rearranged.

Overall, among land plants it seems that there are no strong candidates for gene relocation via direct transposition or translocation. As with *Selaginella*, gene relocations affecting core leptosporangiate ferns were better explained by overlapping inversions (Hasebe & Iwatsuki, 1992; Stein *et al*., 1992; Raubeson & Stein, 1995). In that case, evidence for the first inversion comes from the fact that the orientation of the rRNA operon has flipped and is now transcribed towards the LSC in core leptosporangiate ferns, instead of towards the SSC as seen in nearly all other land plant plastomes. Likewise, overlapping inversions with distinct evolutionary intermediates were shown to be better explanations than transposition/translocation for the suite of genomic rearrangements observed among species in the Oleaceae (Lee *et al*., 2007). It therefore seems prudent to assume that the LSC‐to‐SSC relocation of *ycf2* in all sampled *Isoetes* genomes (Fig. 4) resulted from the pair of overlapping inversions rather than direct transposition/translocation. Currently, *Isoetes* sampling is restricted to species from clades C (*I. malinverniana*) and E (*I. flaccida* and *I. piedmontana*), following the classification of Larsén & Rydin (2016). Sampling of *Isoetes* species from clades A and B, which fall outside of the C + E clade, may identify species having only one of the two inferred inversions, which would provide strong support for the overlapping inversion hypothesis in *Isoetes*.

## Author contributions

JPM planned and designed research. JPM, FG, AT and Y‐LQ collected plant specimens and prepared samples for sequencing. JPM, P‐FM, FG, TPM and RV performed experimental and/or computational analyses. All authors interpreted results. JPM wrote the manuscript, with input from all other authors.

## Supporting information

Please note: Wiley Blackwell are not responsible for the content or functionality of any Supporting Information supplied by the authors. Any queries (other than missing material) should be directed to the *New Phytologist* Central Office.

**Dataset S1** DNA sequence alignments for 51 single‐copy genes, four rRNA genes, *ndhB *+ *psbM *+ *rps7*, and *rps4*.
**Fig. S1** Verification of the direct repeat (DR) orientation in the *Selaginella kraussiana* plastome.**Fig. S2** Structural evolution of the *Selaginella uncinata* plastome.
**Table S1** Sample and sequencing information for lycophyte plastomes.
