# Supplementary material for: Lycophyte plastid genomics: extreme variation in GC, gene and intron content and multiple inversions between a direct and inverted orientation of the rRNA repeat
Source: New Phytol. 2019 Jan 24;222(2):1061–75. doi: 10.1111/nph.15650 (PMC6590440; doi:10.1111/nph.15650)
Supplement: Supplementary file 1 — Dataset S1 DNA sequence alignments for 51 single‐copy genes, four rRNA genes, ndhB + psbM + rps7, and rps4. Fig. S1 Verification of the direct repeat (DR) orientation in the Selaginella kraussiana plastome.Fig. S2 Structural evolution of the Selaginella uncinata plastome. Table S1 Sample and sequencing information for lycophyte plastomes. [file NPH-222-1061-s001.zip › nph15650-sup-0001-SupInfo.pdf]

New Phytologist

***New Phytologist* Supporting Information**

Article title: Lycophyte plastid genomics: extreme variation in GC, gene and intron content and multiple inversions between a direct and inverted orientation of the rRNA repeat

Authors: Jeffrey P. Mower, Peng-Fei Ma, Felix Grewe, Alex Taylor, Todd P. Michael, Robert VanBuren, Yin-Long Qiu

Article acceptance date: 10 December 2018

The following Supporting Information is available for this article:

Fig. S1 Verification of the direct repeat (DR) orientation in the *Selaginella kraussiana* plastome.

Fig. S2 Structural evolution of the *Selaginella uncinata* plastome.

Table S1 Sample and sequencing information for lycophyte plastomes.

Dataset S1 DNA sequence alignments for 51 single-copy genes, 4 rRNA genes,

ndhB+psbM+rps7, and *rps4* (see separate file)

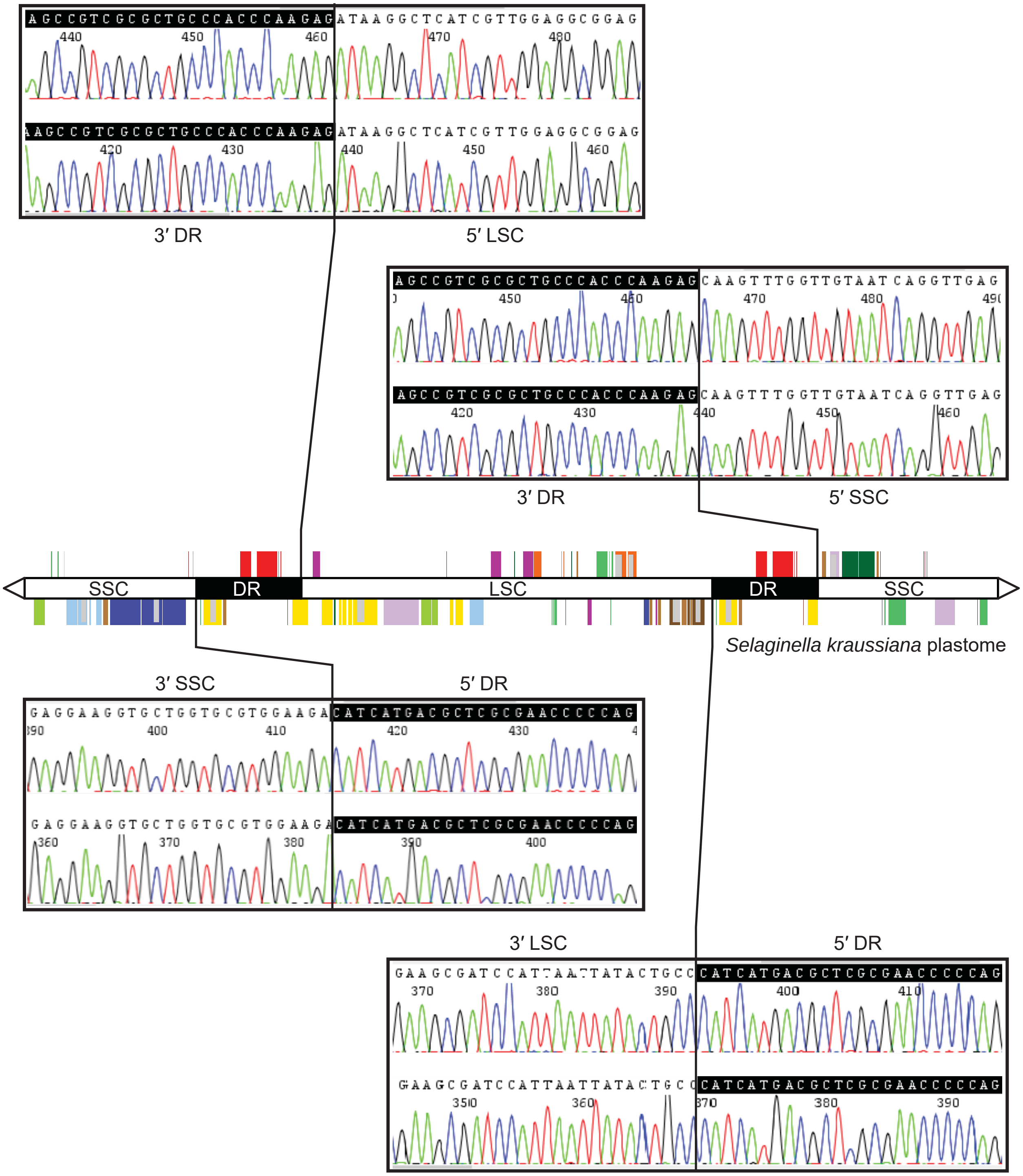

Fig. S1 Verification of the direct repeat (DR) orientation in the *Selaginella kraussiana* plastome. The plastome map is shown, and the large single copy (LSC) region, small single copy (SSC) region (split into two parts connected by arrowheads), and two copies of the DR are labeled. Sanger sequencing results, spanning each DR/LSC and DR/SSC junction, are provided for the forward (top) and reverse (bottom) sequencing direction. Sanger sequence calls from the DR are shown in white text with black background, while sequence calls from the LSC and SSC are shown in black text with white background.

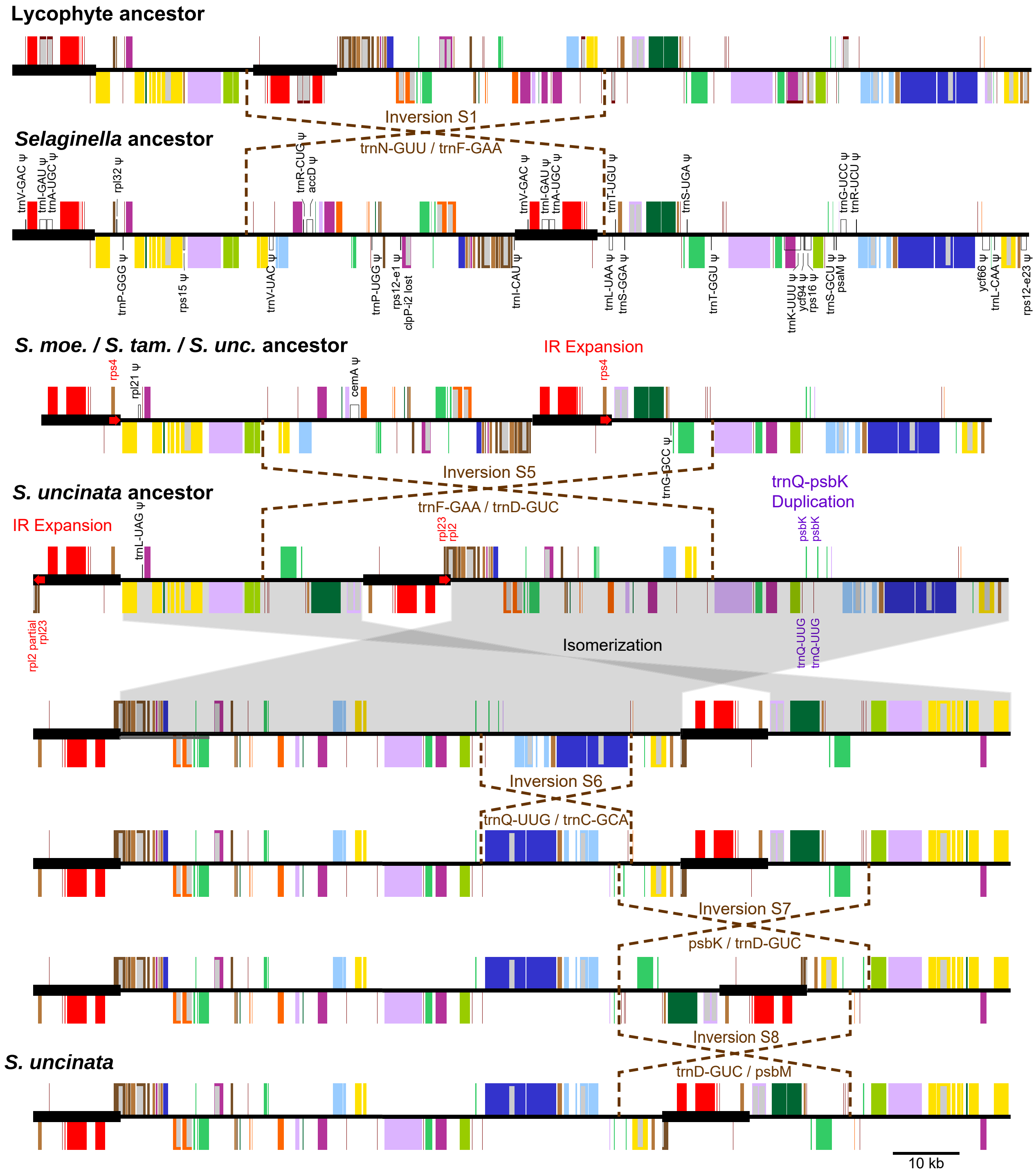

Fig. S2 Structural evolution of the *Selaginella uncinata* plastome. Pseudogenes and lost genes are marked with a ψ and listed in black text. Inversion endpoints are marked by brown dotted lines, and the genes closest to each endpoint are listed in brown text. Inverted repeat (IR) expansions are denoted with red arrows, and genes affected by the IR expansion are listed in red text. A shift between plastome isomers (highlighted in gray) was performed for illustration purposes by inverting the orientation of the small single copy (SSC) region relative to the large single copy (LSC) region. The *trnQ-psbK* duplication is identified with purple text.

Table S1. Sample and sequencing information for lycophyte plastomes

Species	Source information	Voucher, herbarium	Sequencing platform	Library size (bp)	Read len (bp)	Total output (Gb)	Coverage depth [a]	Accession number
Dendrolycopodium obscurum	Lake Erickson, Vilas Co, WI	Qiu 94168, IND	HiSeq 4000	300	2 x 151	12.9	970	MH549637
Diphasiastrum digitatum	Graveyard Fields, NC	none [b]	HiSeq 2500	750	2 x 125	11.0	90	MH549638
Huperzia lucidula	Kemp Natural Resources Station, WI	Qiu 94173, IND	HiSeq 4000	350	2 x 151	13.2	990	MH549639
Isoetes malinverniana	Botanical Garden, Univ. Zurich	Qiu 99050, Z	HiSeq 4000	300	2 x 151	20.8	570	MH549640
Isoetes piedmontana	Rock Grove Church, NC	Grewe FG0001, F	NextSeq	200	2 x 151	17.0	450	MH549641
Lycopodium clavatum	Kemp Natural Resources Station, WI	Qiu 94170, IND	HiSeq 4000	250	2 x 151	8.3	1200	MH549642
Selaginella kraussiana	SRA: SRR2037123	none [c]	HiSeq 3000	150	2 x 101	14.6	400	MH549643
Selaginella lepidophylla	Chihuahuan Desert, USA	none [d]	PacBio RSII	N/A	~30000	19.3	1100	MK089531

N/A, not applicable.

[a] Based on a subset of 20 million reads for all Illumina HiSeq and NextSeq data sets

[b] Too few individuals at collection site to make voucher. Identify verified by blast: 100% similar to EU749489 and 99.9% similar to MG560490

[c] No voucher listed in SRA. Identity verified by blast: 100% similar to KT161498 and 99.8% similar to KY023058

[d] No voucher collected. Identity verified by blast (VanBuren et al. 2018).

References

VanBuren R, Wai CM, Ou S, Pardo J, Bryant D, Jiang N, Mockler TC, Edger P, Michael TP. 2018. Extreme haplotype variation in the desiccation-tolerant clubmoss *Selaginella lepidophylla*. *Nature Communications* **9**: 13.
